# Knowledge of and Attitudes to Influenza Vaccination among Community Pharmacists in Catalonia (Spain). 2013–2014 Season: A Cross Sectional Study

**DOI:** 10.3390/ijerph14070756

**Published:** 2017-07-11

**Authors:** Diana Toledo, Núria Soldevila, Rafel Guayta-Escolies, Pau Lozano, Pilar Rius, Pilar Gascón, Angela Domínguez

**Affiliations:** 1 CIBER Epidemiología y Salud Pública (CIBERESP), 28029 Madrid, Spain; nuriasolde_@hotmail.com (N.S.); angela.dominguez@ub.edu (A.D.); 2 Departament de Medicina, Universitat de Barcelona, 08036 Barcelona, Spain; 3 Unitat de Projectes i Recerca, Consell de Col·legis Farmacèutics de Catalunya, 08009 Barcelona, Spain; rguayta@ccfc.cat (R.G.-E.); plozano@ccfc.cat (P.L.); prius@ccfc.cat (P.R.); pgascon@ccfc.cat (P.G.)

**Keywords:** community pharmacists, influenza vaccination, coverage, knowledge, attitudes

## Abstract

Annual recommendations on influenza seasonal vaccination include community pharmacists, who have low vaccination coverage. The aim of this study was to investigate the relationship between influenza vaccination in community pharmacists and their knowledge of and attitudes to vaccination. An online cross-sectional survey of community pharmacists in Catalonia, Spain, was conducted between September and November 2014. Sociodemographic, professional and clinical variables, the history of influenza vaccination and knowledge of and attitudes to influenza and seasonal influenza vaccination were collected. The survey response rate was 7.33% (506 out of 6906); responses from 463 community pharmacists were included in the final analyses. Analyses were performed using multivariable logistic regression models and stepwise backward selection method for variable selection. The influenza vaccination coverage in season 2013–2014 was 25.1%. There was an association between vaccination and correct knowledge of the virus responsible for epidemics (adjusted Odds Ratio (aOR) = 1.74; 95% CI 1.03–2.95), recommending vaccination in the postpartum (aOR = 3.63; 95% CI 2.01–6.55) and concern about infecting their clients (aOR = 5.27; 95% CI 1.88–14.76). In conclusion, community pharmacists have a very low influenza vaccination coverage, are not very willing to recommend vaccination to all their customers but they are concerned about infecting their clients.

## 1. Introduction

Influenza is the most common vaccine-preventable disease and is recognized to cause serious illness, principally in the elderly, children aged <2 years, pregnant women and people with underlying high-risk medical conditions [1]. Vaccination is a particularly effective public health action against influenza as it prevents death and illness and reduces hospital admissions [2,3]. Improving vaccination coverage to prevent the spread of influenza is a major goal in many developed countries [4].

However, vaccination rates of people with criteria for vaccination remain low due to a complex mix of factors, some of which are linked to care organization models and healthcare workers’ (HCWs) knowledge of and attitudes to vaccination. HCW behaviors and ideas about vaccination may have a strong influence on coverage rates in the general population [4,5,6].

The United States (U.S.) Advisory Committee on Immunization Practices recommends seasonal influenza vaccination to HCWs, including community and hospital pharmacists [2]. In Catalonia, as in the rest of Spain, influenza vaccination is also recommended for HCWs and it’s free of charge [7]. Despite recommendations to improve influenza vaccination coverage among HCWs, they remain below global targets [3,4,5].

Most studies on knowledge, beliefs and attitudes to influenza vaccination have focused on hospital and primary care HCWs [5,6,8]. Hospital HCWs usually have higher influenza coverages than primary or community HCWs, especially in countries where vaccination is mandatory [9]; in this setting, hospital pharmacists have a similar coverage to that of physicians and nurses [10,11]. There are few studies on influenza vaccination coverage in community pharmacists (CPs), but it seems to be low [11,12,13]. A Catalonian study carried out in CPs in Lleida found a coverage of <20% [14]. Barriers to vaccination of HCWs include misconceptions or lack of knowledge, the potential severity of the disease and the perception that the vaccine is not really effective [11,12,13]. Positive facilitators may include previous vaccination behavior, the desire to protect oneself, family or patients, and confidence in the effectiveness of the vaccine [5,6,8,11,12].

In the last decade, countries like U.S., France, Switzerland, U.K., Portugal, Japan and Canada have introduced new measures to increase influenza vaccination coverage in the population by using the role of CPs as advocates, educators, facilitators and immunizers [4,15,16,17]. In Catalonia, as in Spain, the CPs’ role is focused on advocacy and education on vaccination [18].

CPs are the most accessible HCWs for the general population and are in continuous contact with persons with influenza and those at risk of infection and of being a source of transmission to persons without influenza attending pharmacies [12]. Vaccination of CPs is important to prevent the spread of the virus. In addition, perceptions of and attitudes to influenza vaccination may influence their recommendations to patients and may be a marker for the evaluation of occupational risks. The aim of this study was to investigate the associations between influenza vaccination of CPs and their knowledge of and attitudes to this preventive measure.

## 2. Materials and Methods

### 2.1. Design

A cross-sectional study was conducted using an anonymous online survey of CPs in Catalonia, a Spanish region with a population of 7.5 million. The survey was active from 1 September to 30 November 2014.

### 2.2. Study Subjects

Potential study subjects were the 6906 CPs registered in any of the four Official Colleges of Pharmacists in Catalonia—Barcelona (COFB), Girona (COFGi), Lleida (COFLl) and Tarragona (COFT). All CPs registered have credentials to log in into their college intranet, which is used as an informative and communicative tool by pharmacists and their representatives. All CPs were invited to participate in the survey through a written invitation from the head of the Council of Colleges of Pharmacists of Catalonia published in the intranet corresponding to the demarcation. Access to the survey was through a direct link located on the intranet. The survey was active for 91 days and three reminders were included in the monthly newsletter of the Colleges of Pharmacists.

### 2.3. Questionnaire Design

The questionnaire was designed based on the review of instruments used in scientific publications related to the study topic, especially the questionnaire used by Kraut et al. and its adaptation to a study conducted in Spain [3,19]. The questions were adapted to the specific circumstances of pharmacists’ professional tasks within the Spanish National Health System, ensuring conceptual and semantic equivalence.

Two pilot studies with 10 participants were conducted. The mean response time was 7.3 min. Survey information was collected by the www.encuestafacil.com platform. Community pharmacists were invited to participate in confidence, no personal identifiers were collected and no incentives offered. The study was approved by the Council of Colleges of Pharmacists in Catalonia (Working Group of the Services Portfolio, Council of Colleges of Pharmacists) on 14 June 2014.

### 2.4. Study Measures

The final questionnaire consisted of five sections: (a) sociodemographic and professional variables: age (<34 years, 35–44 years, 45–54 years, ≥55 years), sex (female/male), cohabitation status (person(s) <15 years, person(s) >65 years, chronically ill person(s)), professional category (titular pharmacist, assistant or substitute pharmacist), years of work (≤9 year, 10–29 year, ≥30 year) and location of workplace (according to the Spanish Institute of Statistics [20], rural and intermediate ≤10,000 and urban >10,000); (b) Clinical information: Current condition or disease included in the influenza vaccination recommendations (Yes/No), belonging to a risk group (Yes/No); (c) History of influenza vaccination: in the current season (2013–2014) (Yes/No) and the three previous seasons (Yes/No); (d) Knowledge of and recommendations on influenza and seasonal influenza vaccination: Knowledges of influenza and seasonal influenza vaccination (correct/incorrect), recommendations to target population (people with chronic disorders, immunosuppressed people or pregnant women) (Yes/No) and any training in influenza in last five years (Yes/No); (e) Attitudes to influenza and seasonal influenza vaccination and opinion on the recommendations made by health authorities during the pandemic, evaluated on a Likert scale with five categories (totally agree, agree quite a lot, neither agree or disagree, disagree quite a lot, and totally disagree.

### 2.5. Statistical Analysis

The CPs who partially completed the survey and those with contraindications to vaccination or where vaccination was indicated due to a medical risk condition were excluded from the study.

The response categories for questions related to attitudes to influenza and seasonal influenza vaccination and opinion on the recommendations made by health authorities during the pandemic, evaluated on a Likert scale with five categories were dichotomized into “agree” (including “agree” and “strongly agree”) and “disagree” (including “neither agree nor disagree”, “disagree” and “strongly disagree”).

A bivariate analysis was made comparing the responses of CPs vaccinated and unvaccinated in the 2013–2014 influenza season. Sociodemographic variables and variables on the vaccination history, knowledge of and recommendations and attitudes to influenza and influenza vaccination were performed using unadjusted logistic regression to generate crude odds ratios. A two-sided *p*-value <0.05 was considered to indicate a statistically significant difference.

Independent variables were checked for multi-collinearity: collinearity was found between the variables “years of work” and “age”, and therefore it was decided to omit the variable “years of work” from the two final models. Two multivariable models were constructed: one for knowledge and one for attitudes; sociodemographic variables were considered as variables of adjustment in both models.

A multivariable analysis was performed using logistic regression with stepwise backward selection of variables and a cut-off point of <0.2 to estimate the association between CPs vaccinated status and knowledge of influenza and attitudes towards influenza vaccination. The goodness-of-fit of the two models was evaluated using the Hosmer-Lemeshow test. The analysis was performed using SPSS version 23 (SPSS Inc., Chicago, IL, USA).

## 3. Results

**Figure 1:**
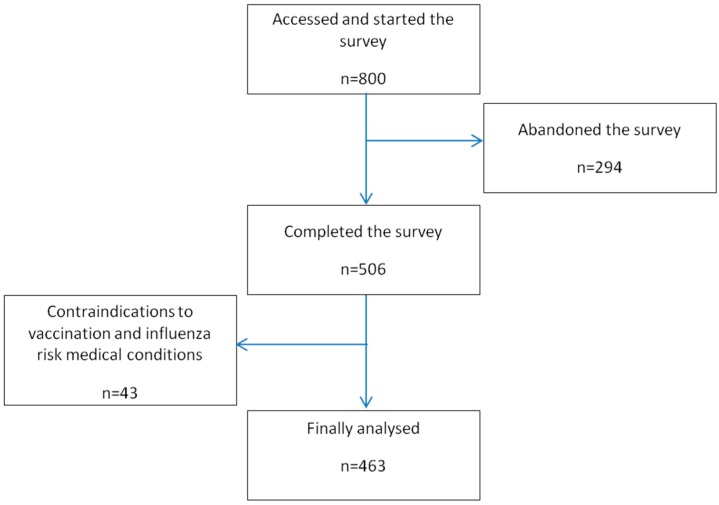
Flow chart of CPs’ access to and participation in the survey.

The cooperation rate (proportion of CPs who accessed the survey) was 11.58% (800) and the response rate (proportion of CPs who completed the survey) was 7.33% (506). Of the CPs who completed the survey, 9 were excluded due to contraindications to influenza vaccination and 34 in whom vaccination was indicated due to risk medical conditions (Figure 1).

Therefore, data on 463 CPs were finally analyzed: 33.3% (154) were aged 45–54 years, 77.8% (360) were female, 60.5% (280) were titular pharmacists, 67.0% (310) reported a work experience of 10–29 years, 70.0% (324) worked in pharmacies located in urban areas, and 36.1% (167) reported having received the vaccine in any of the last three seasons.

The proportion of CPs who responded that they would recommend influenza vaccinations for pregnant women ranged from 29.8% (138) during the first trimester to 44.1% (204) in the second and third trimester and 30.9% (143) after childbirth. Recommendations were around 95% for people aged ≥65 years and 63.7% for immunosuppressed people (Table S1).

The influenza vaccination coverage in the 2013–2014 season was 25.1% (116) and increased with age. Table 1 shows the highest coverage was in the ≥55 years age group (*p* = 0.001); males were vaccinated more than females (*p* < 0.001), vaccination was less in substitute pharmacists than titular pharmacists (*p* = 0.04) and in cohabiting with persons aged ≥65 years (*p* < 0.001).

Table 2 and Table 3 shows the relationship between influenza vaccination in 2013–2014 season and knowledge of and recommendations about influenza and seasonal influenza vaccination. Table 3 summarize the results of the multivariable analysis, factors associated independently with vaccination were age ≥55 years (aOR = 4.42; 95% CI 1.43–13.71), male sex (aOR = 2.95; 95% CI 1.72–5.04), being an assistant pharmacist (aOR = 2.61; 95% CI 1.17–5.84), cohabitation with person aged >65 years (aOR = 2.38; 95% CI 1.25–4.55), had correct responses to knowledge of the virus responsible for epidemics (aOR = 1.74; 95% CI 1.03–2.95), and more frequently recommended vaccination to women after childbirth (aOR = 3.63, 95% CI 2.01–6.45). In contrast, among those CPs who received any specific training on influenza in the last 5 years the odds for influenza vaccination were lower, compared to those who did not (aOR 0.53; 95% CI 0.30–0.94). The *p*-Value for the Hosmer-Lemeshow goodness-of-fit statistic was 0.77.

Table 4 shows the distribution of attitudes to influenza and influenza vaccination in vaccinated and unvaccinated CPs in the 2013–2014 season. The association between vaccination coverage of CPs according to their attitudes to influenza and vaccination is shown in Table 5.

The closest association was with concern about infecting their clients (aOR = 5.27; 95% CI 1.88–14.76) and seasonal vaccination in all the three preceding seasons (aOR = 89.50; 95% CI 32.85–243.86). Meanwhile, CPs who received recommendations on vaccination from their physician or from the College of Pharmacists were less frequently vaccinated than those who received no recommendations (aOR = 0.09; 95% CI 0.03–0.23; 0.31; 95% CI 0.13–0.74), respectively. The *p*-value for the Hosmer-Lemeshow goodness-of-fit statistic was 0.94.

## 4. Discussion

This study examined vaccination rates among CPs in Catalonia and identified perceptions and attitudes that might influence vaccination. Vaccination coverage in Catalonian CPs in the 2013–2014 influenza season was 25.1%, higher than the 19.8% found in Lleida, but well below the coverage in primary care and hospital HCWs in Spain, which ranged between 38% and 50.7% [5,21]. It was also below the coverage in CPs in other Western countries, which ranged between 42% and 75% [10,11,12,22] and the coverage in hospital pharmacists of >88% [10,11].

A possible explanation is that, in countries like the U.S., some pharmacy owners require their workers to be vaccinated or offer incentives for vaccination [11,23]. In Spain, vaccination is recommended in health professionals in agreement with the criteria of national and international organizations [8,18]. However, there is no regulation that requires CPs to ensure their employees are vaccinated.

In line with some authors [5,14,24] we found that a history of vaccination in previous seasons was one factor most-strongly associated with the probability of vaccination in the latest season. In our study, the CPs vaccinated were shown to have correct knowledge of the virus causing epidemics, although there were no differences between vaccinated and unvaccinated CPs with respect to knowledge of the vaccine or of other aspects of influenza infection.

We found no studies on CPs’ knowledge of influenza and influenza vaccination. Therefore, we were unable to compare these results. However, studies in other health professionals, including physicians and nurses, have found that a low level of knowledge of these aspects may influence the decision of professionals to be vaccinated or not [19,25].

Only 29.8% of all CPs surveyed reported any specific training on influenza in the last five years and 79.7% of these were not vaccinated, possibly because the final decision on vaccination may be influenced by factors identified as barriers to vaccination [26]. Our study focused on perceptions and attitudes to influenza and vaccination.

A U.S. study of the perceptions and attitudes of pharmacists to vaccination found that CPs were more likely to believe they were at lower risk of becoming ill due to influenza than hospital, academic or clinical pharmacists [11]. Our results showed no association with these statements but did find an association between the vaccination of pharmacists and concern about infecting their clients. The negative results on vaccination and having received some type of recommendation from general practitioners or the College of Pharmacists might suggest that CPs do not consider themselves at risk of becoming ill or that the messages incentivizing vaccination that they receive are not adequate. Education on the characteristics of influenza and influenza vaccines are important aspects that should be considered for training and education campaigns on influenza and its prevention.

Community pharmacists knowledge and perceptions of and attitudes to influenza and vaccination may be important in determining and predicting vaccination in this group and their possible impact on vaccination coverage of the population, especially in people with risk conditions to which they provide special care [17,27]. The CP is, in fact, the only health professional in constant and close contact with all citizens, sick or not, and who may be accessed without an appointment [4,17].

Our study found no association between vaccination of CPs and recommendations to vaccinate in people aged ≥65 years, with chronic illnesses, or pregnant women, unlike Dolan et al. [22], who found that 73% of vaccinated CPs recommend influenza vaccination for pregnant women. However, we found an association between the vaccinations of CPs and recommending the vaccine to women in the postpartum, comparable to the results shown by Dominguez et al. in a sample of primary HCWs [5]. The low level of recommendation of vaccination during pregnancy may be due to the lack of knowledge of the safety of the vaccine during pregnancy and its indication in any trimester, and to a possible lack of knowledge on the fact that influenza may trigger serious events during pregnancy.

Surveys of CPs often have lower response rates than those made in physicians and nurses, in whom response rates of between 36.2% and 73% have been obtained [5,28]. This may be because CPs work outside hospitals and medical centers, where preventive activities are continuous and this could lead to a lower appreciation of being in an epidemic environment.

The low response rate in our study (7.33%) could be a limitation, but similar response rates were obtained by Atkins et al. [16] in a survey of London CPs (5%) and by Dolan et al. [22] among members of the American Society of Pharmacy (8%); both studies included a large number of CPs. However, the response rate in smaller studies that sent the link to the survey by email was greater. The response rate was 43.1% in Lleida, Spain and 24% in members of the societies of pharmacists specialized in infectious diseases in the U.S. [14,23]. Unfortunately, it was not possible to determine the profile of non-responders due to the scarce and diverse data in the 294 incomplete surveys.

The access route of the survey (intranet) could have led to a response bias and to a low response rate, possibly related to the individual use of the College of Pharmacists intranet, even though this is a communication route used by health authorities to provide news and updates to CPs. However, the response rate was similar to those of other studies using the same access route.

The main strength of this study is that it provides information on influenza vaccination coverage in a relatively-unstudied group. In addition, the study was carried out in a Spanish region with one of the largest number of registered CPs. Another strength is that CPs with contraindications to vaccination or who had high-risk chronic diseases were excluded from the analysis.

## 5. Conclusions

In conclusion, our results show that CPs had a very low rate of influenza vaccination coverage in spite of expressing concerns about infecting their clients. In addition, our study showed that only a fraction of the CPs were willing to recommend influenza vaccination for some of the risk groups in whom vaccination is indicated and a low rate of vaccination related to the recommendation to be vaccinated by the health authorities. The discourse of campaigns to incentivize the vaccination of CPs should be rethought and adapted to design specific training programs on influenza and influenza vaccination that empower their role in the prevention of infectious diseases, establishing incentives and developing polices such as those that exist in other countries to increase vaccine coverages in this group and, possible, help to raise vaccination rates.

## Figures and Tables

**Table 1:** Distribution of influenza vaccinated and unvaccinated community pharmacists in the 2013–2014 season by demographic and professional characteristics.

Demographic and Professional Characteristics	Vaccinated *n* = 116	Unvaccinated *n* = 347	Crude OR (95% CI)	*p*-Value
**Age group**				
≤34 years	6 (10.5%)	51 (89.5%)	1	
35–44 years	32 (21.8%)	115 (78.2%)	2.37 (0.93–6.01)	0.07
45–54 years	38 (24.7%)	116 (75.3%)	2.78 (1.11–7.00)	0.03
≥55 years	40 (38.1%)	65 (61.9%)	5.23 (2.06–13.30)	0.001
**Sex**				
Female	75 (20.8%)	285 (79.2%)	1	
Male	41 (39.8%)	62 (60.2%)	2.51 (1.57–4.02)	<0.001
**Professional category**				
Titular pharmacist	76 (27.1%)	204 (72.9%)	1	
Assistant pharmacist	18 (31.0%)	40 (69.0%)	1.21 (0.65–2.24)	0.55
Substitute pharmacist	22 (17.6%)	103 (82.4%)	0.57 (0.34–0.97)	0.04
**Years of work**				
≤9 years	8 (14.0%)	49 (86.0%)	1	
10–29 years	74 (23.9%)	236 (76.1%)	1.92 (0.87–4.24)	0.11
≥30 years	34 (35.4%)	62 (64.9%)	3.36 (1.43–7.91)	0.01
**Type of population**				
Rural	32 (26.7%)	88 (73.3%)	1	
Urban	78 (24.1%)	246 (75.9%)	0.87 (0.54–1.41)	0.57
**Living with children aged ≤15 years**	50 (23.8%)	160 (76.2%)	0.89 (0.58–1.35)	0.57
**Living with persons with chronic disease**	17 (33.3%)	34 (66.7%)	1.58 (0.85–2.95)	0.15
**Living with persons aged ≥65 years**	30 (42.9%)	40 (57.1%)	2.68 (1.58–4.55)	<0.001

**Table 2:** Distribution of influenza vaccinated and unvaccinated community pharmacists in the 2013–2014 season according to knowledge of and recommendations about influenza and influenza vaccination.

Knowledge and Recommendations	Vaccinated *n* = 116	Unvaccinated *n* = 347	Crude OR (95% CI)	*p*-Value
**Knowledge of the strains contains in the influenza vaccine**				
Incorrect	28 (19.3%)	117 (80.7%)	1	
Correct	88 (27.7%)	230 (72.3%)	1.60 (0.99–2.58)	0.06
**Knowledge of the virus responsible for epidemics**				
Incorrect	31 (18.5%)	137 (81.5%)	1	
Correct	85 (28.8%)	210 (71.2%)	1.79 (1.13–2.85)	0.01
**Knowledge about the influenza incubation period**				
Incorrect	74 (26.7%)	203 (73.3%)	1	
Correct	42 (22.6%)	144 (77.4%)	0.80 (0.52–1.24)	0.32
**Knowledge about influenza transmission**				
Incorrect	38 (21.7%)	137 (78.3%)	1	
Correct	78 (27.1%)	210 (72.9%)	1.34 (0.86–2.09)	0.20
**I recommend the vaccine to pregnant women in their first trimester**				
No	76 (23.4%)	249 (76.6%)	1	
Yes	40 (29.0%)	98 (71.0%)	1.34 (0.85–2.09)	0.20
**I recommend the vaccine to pregnant women in their second or third trimester**				
No	65 (25.1%)	194 (74.9%)	1	
Yes	51 (25.0%)	153 (75.0%)	1.00 (0.65–1.52)	0.98
**I recommend the vaccine to post-partum women**				
No	62 (19.4%)	258 (80.6%)	1	
Yes	54 (37.8%)	89 (62.2%)	2.53 (1.63–3.91)	<0.001
**I recommend the vaccine to people with chronic disorders**				
No	3 (12.5%)	21 (87.5%)	1	
Yes	113 (25.7%)	326 (74.3%)	2.43 (0.71–8.29)	0.16
**I recommend the vaccine to immunosuppressed people**				
No	35 (20.8%)	133 (79.2%)	1	
Yes	81 (27.5%)	214 (72.5%)	1.44 (0.92–2.26)	0.12
**Any specific training in the influenza in the last five years**				
No	88 (27.1%)	237 (72.9%)	1	
Yes	28 (20.3%)	110 (79.7%)	0.69 (0.42–1.11)	0.12

**Table 3:** Influenza vaccination coverage in community pharmacists in the 2013–2014 season according to knowledge of and recommendations about influenza and influenza vaccination.

Factors Associated	Adjusted OR (95% CI)	*p*-Value
**Age group**		
≤34 years	1	
35–44 years	1.99 (0.72–5.53)	0.19
45–54 years	2.13 (0.73–6.24)	0.17
≥55 years	4.42 (1.43–13.71)	0.01
**Sex.** Male	2.95 (1.72–5.04)	<0.001
**Professional category**		
Titular pharmacist	1	
Assistant pharmacist	2.61 (1.17–5.84)	0.02
Substitute pharmacist	0.99 (0.51–1.92)	0.96
**Cohabitation with person aged >65 years**	2.38 (1.25–4.55)	0.01
**Correct knowledge of the virus responsible for epidemics**	1.74 (1.03–2.95)	0.04
**Correct knowledge about the influenza incubation period**	0.67 (0.40–1.11)	0.12
**I recommend the vaccine to pregnant women in their second or third trimester**	0.63 (0.35–1.14)	0.13
**I recommend the vaccine to post-partum women**	3.63 (2.00–6.55)	<0.001
**Any specific training on influenza in the last five years**	0.53 (0.30–0.94)	0.03

**Table 4:** Distribution of influenza vaccinated and unvaccinated community pharmacists in the 2013–2014 season according to attitudes towards influenza vaccination.

Attitudes	Vaccinated *n* = 116	Unvaccinated *n* = 347	Crude OR (95% CI)	*p*-Value
**My general practitioner motivated me to get vaccinated**				
No	90 (45.7%)	107 (54.3%)	1	
Yes	26 (9.8%)	240 (90.2%)	0.13 (0.08–0.21)	<0.001
**The College of Pharmacists recommended vaccination to me**				
No	65 (46.4%)	75 (53.6%)	1	
Yes	51 (15.8%)	272 (84.2%)	0.22 (0.14–0.34)	<0.001
**Concern about infection at work**				
No	14 (5.8%)	227 (94.2%)	1	
Yes	102 (45.9%)	120 (54.1%)	13.78 (7.56–25.13)	<0.001
**Influenza can be a serious illness**				
No	36 (19.5%)	149 (80.5%)	1	
Yes	80 (28.8%)	198 (71.2%)	1.67 (1.07–2.62)	0.02
**Vaccination is effective in preventing influenza and its complications**				
No	8 (7.1%)	105 (92.9%)	1	
Yes	108 (30.9%)	242 (69.7%)	5.86 (2.76–12.45)	<0.001
**Concern about becoming ill**				
No	20 (8.7%)	209 (91.3%)	1	
Yes	96 (41.0%)	138 (59.0%)	7.27 (4.29–12.32)	<0.001
**Concern about infecting my family**				
No	16 (7.9%)	187 (92.1%)	1	
Yes	100 (38.5%)	160 (61.5%)	7.31 (4.14–12.90)	<0.001
**Concern about infecting my clients**				
No	21 (9.3%)	204 (90.7%)	1	
Yes	95 (39.9%)	143 (60.1%)	6.45 (3.84–10.84)	<0.001
**Vaccination of pharmacists workers is important**				
No	21 (9.3%)	204 (90.7%)	1	
Yes	99 (85.3%)	158 (45.5%)	6.97 (3.99–12.15)	<0.001
**Vaccination is effective because it reduces the costs related to influenza in patients**				
No	14 (9.3%)	136 (90.7%)	1	
Yes	102 (32.6%)	211 (67.4%)	4.70 (2.58–8.55)	<0.001
**Vaccinating high-risk subjects every influenza season is effective in reducing the complications of influenza**				
No	3 (8.1%)	34 (91.9%)	1	
Yes	113 (26.5%)	313 (73.5%)	4.09 (1.23–13.58)	0.02
**Vaccination is the most important measure in preventing influenza infection**				
No	8 (7.2%)	103 (92.8%)	1	
Yes	108 (93.1%)	244 (70.3%)	5.70 (2.68–12.12)	<0.001
**Activities carried out during 2009–2010 were adjusted to the evolution of the pandemic**				
No	11 (16.2%)	57 (83.8%)	1	
Yes	105 (26.6%)	290 (73.4%)	1.88 (0.95–3.71)	0.07
**Seasonal vaccination in all 3 preceding seasons**				
No	22 (6.3%)	330 (93.8%)	1	
Yes	94 (84.7%)	17 (15.3%)	82.94 (42.31–162.59)	<0.001

**Table 5:** Influenza vaccination coverage in community pharmacists in the 2013–2014 season according to attitudes towards influenza vaccination.

Factors Associates	Adjusted OR (95% CI)	*p*-Value
Cohabitation with person with chronic disease	2.89 (0.94–8.86)	0.07
My general practitioner motivated me to get vaccinated	0.09 (0.03–0.23)	<0.001
The College of Pharmacists recommended vaccination to me	0.31 (0.13–0.74)	0.01
Concern about infection at work	2.46 (0.87–6.92)	0.09
Concern about infecting my clients	5.27 (1.88–14.76)	0.002
Seasonal vaccination in all 3 preceding seasons	89.50 (32.85–243.86)	<0.001

## Supplementary Materials

The following are available online at www.mdpi.com/1660-4601/9/7/756/s1, Table S1: Distribution of characteristics of all pharmacists finally analyzed.
