# Supplementary material for: Knowledge of and Attitudes to Influenza Vaccination among Community Pharmacists in Catalonia (Spain). 2013–2014 Season: A Cross Sectional Study
Source: Int J Environ Res Public Health. 2017 Jul 11;14(7):756. doi: 10.3390/ijerph14070756 (PMC5551194; doi:10.3390/ijerph14070756)
Supplement: Supplementary file 1 [file ijerph-14-00756-s001.pdf]

Table S1. Distribution of characteristics of all pharmacists finally analyzed

Characteristics	Community pharmacist (*n* = 463)
**Age group**	
≤34 years	57 (12.3%)
35–44 years	147 (31.7%)
45–54 years	154 (33.3%)
≥55 years	105 (22.7%)
**Sex**	
Female	360 (77.8%)
Male	103 (22.2%)
**Professional category**	
Titular pharmacist	280 (60.5%)
Assistant pharmacist	58 (12.5%)
Substitute pharmacist	125 (27.0%)
**Years of work**	
≤ 9 years	57 (12.3%)
10–29 years	310 (67.0%)
≥ 30 years	96 (20.7%)
**Type of population**	
Rural	120 (25.9%)
Urban	324 (70.0%)
**Seasonal vaccination 2013/14**	
Yes	116 (25.1%)
**Seasonal vaccination in any of the three preceding seasons**	
Yes	167 (36.1%)
**Seasonal vaccination in all three preceding seasons**	
Yes	111 (24.0%)
**Vaccination with pandemic vaccine 2009-2010**	
Yes	167 (36.1%)
**I recommend the vaccine to pregnant women in their first trimester**	
Yes	138 (29.8%)
**I recommend the vaccine to pregnant women in their second or third trimester**	
Yes	204 (44.1%)
**I recommend the vaccine to post-partum women**	
Yes	143 (30.9%)
**I recommend the vaccine to people with chronic disorders**	
Yes	439 (94.8%)
**I recommend the vaccine to immunosuppressed people**	
Yes	295 (63.7%)
**I recommend the vaccine to persons aged > 65 years**	
Yes	440 (95.0%)
